# A Novel Chromatographic Method to Assess the Binding Ability towards Dicarbonyls

**DOI:** 10.3390/molecules28145341

**Published:** 2023-07-11

**Authors:** Angelica Artasensi, Emanuele Salina, Laura Fumagalli, Luca Regazzoni

**Affiliations:** Department of Pharmaceutical Sciences, University of Milan, via Mangiagalli 25, 20133 Milan, Italy; angelica.artasensi@unimi.it (A.A.); emanuele.salina1@studenti.unimi.it (E.S.); laura.fumagalli@unimi.it (L.F.)

**Keywords:** high performance liquid chromatography, binding assay, dicarbonyls, methylglyoxal, benzylglyoxal

## Abstract

Human exposure to dicarbonyls occurs via ingestion (e.g., food), inhalation (e.g., electronic cigarettes) and dysregulation of endogenous metabolic pathways (e.g., glycolysis). Dicarbonyls are electrophiles able to induce carbonylation of endogenous substrate. They have been associated with the onset and progression of several human diseases. Several studies have advocated the use of dicarbonyl binders as food preservatives or as drugs aimed at mitigating carbonylation. This study presents the setup of an easy and cheap assay for the screening of selective and potent dicarbonyl binders. The method is based on the incubation of the candidate molecules with a molecular probe. The activity is then determined by measuring the residual concentration of the molecular probe over time by liquid chromatography (LC). However, the naturally occurring dicarbonyls (e.g., glyoxal, methylglyoxal) are not appealing as probes since they are hard to separate and detect using the most popular LC variants. Benzylglyoxal (BGO) was therefore synthesized and tested, proving to be a convenient probe that allows a direct quantification of residual dicarbonyls by reversed phase LC without derivatization. The method was qualified by assessing the binding ability of some molecules known as binders of natural occurring dicarbonyls, obtaining results consistent with literature.

## 1. Introduction

Glyoxal (GO) and methylglyoxal (MGO) are the two most representative molecules among the naturally occurring dicarbonyls. Since such molecules are typically produced by oxidative degradation of lipids and sugars, human exposure to dicarbonyls can occur either via ingestion (e.g., food) or inhalation (e.g., electronic cigarettes) [1,2,3,4]. Dicarbonyls are also produced by endogenous metabolic pathways (e.g., glycolysis, protein glycation) [5,6,7,8]. Their concentration is therefore finely regulated by a set of enzymes, including glyoxalases, reductases, and dehydrogenases [9,10,11,12].

An accumulation of dicarbonyls can be due to an overload of the exogenous intake of such compounds or to the depletion and dysregulation of endogenous substrates or enzymes that regulate their concentration [2,13,14]. Dicarbonyls are electrophiles able to covalently bind biomolecules such as DNA and proteins in order to generate the so-called advanced glycation end products (AGEs) [15,16,17]. An uncontrolled accumulation of AGEs (i.e., carbonylation) is correlated with the onset and progression of several human diseases [18,19].

The most likely explanation of the pathogenic role of AGEs is the alteration of the natural functions of biological molecules modified by dicarbonyls, along with an increased oxidative stress and immune response.

For this reason, the development of dicarbonyl binders has been advocated as a potential strategy to develop new food preservatives or new drugs for the mitigation of the accumulation of AGEs in humans. To date, few molecules have been already reported as dicarbonyl binders [20,21,22,23]. The studies were performed by using an array of diverse analytical techniques, including liquid chromatography, UV and fluorescence spectroscopy, or mass spectrometry. However, it is hard to setup a robust, universal method for the screening of candidate binders, especially if the molecules have different structures and are detectable only by using specific detectors.

For this reason, a convenient chromatographic assay was successfully applied to study molecules able to interact with another class of carbonyl toxicants involved in human disease (i.e., α,β-unsaturated aldehydes). This technique was not only used for the identification of hit compounds (e.g., carnosine), but also for further studies aimed at structure optimization [24]. The assay was built around a naturally occurring molecule (hydroxynonenal, HNE), which was identified as an analytically convenient probe that averages the reactivity of the whole class of α,β-unsaturated aldehydes. HNE is analytically convenient as it is stable and can be easily quantified by means of liquid chromatography (LC) with UV detection. Upon incubation with HNE, the binders can be identified as compounds able to determine a decrease over time of the residual concentration of HNE, as measured by LC-UV.

Unfortunately, GO, MGO, or the other naturally occurring dicarbonyls are not suitable for the development of a similar assay. In fact, they are, on the one hand, not detectable with the most popular detectors for chromatography (e.g., UV or MS). On the other hand, they are not retained by the most common chromatography variants owing to their hydrophilicity. For these reasons, the analysis of such compounds by LC typically requires derivatization [1,3,4,25,26,27] or other chemical reactions (e.g., oxidation) aimed at converting the analytes into detectable molecules [28].

Nevertheless, the analytical strategies relying on derivatization before quantification are not exempt from risks. In fact, the candidate binders incubated with the molecular probe can affect the derivatization yield. Alternatively, the chemicals used for derivatization can break the adducts between the molecular probe and its binder. These are just two examples of potential side reactions leading to an underestimation or overestimation of the residual amount of the molecular probe, with the risk of identifying false positives or false negatives during the screening.

To overcome such limitations, an alternative analytical strategy is herein reported. A UV responsive surrogate of naturally occurring dicarbonyls (i.e., benzylglyoxal, BGO) was synthesized and used as a molecular probe to assess the binding activity of dicarbonyl binders without derivatization. This strategy allowed us to setup a simple and cost-effective chromatographic assay that was validated by testing molecules with already established binding activity towards MGO and GO.

## 2. Results and Discussion

**Figure 1:**
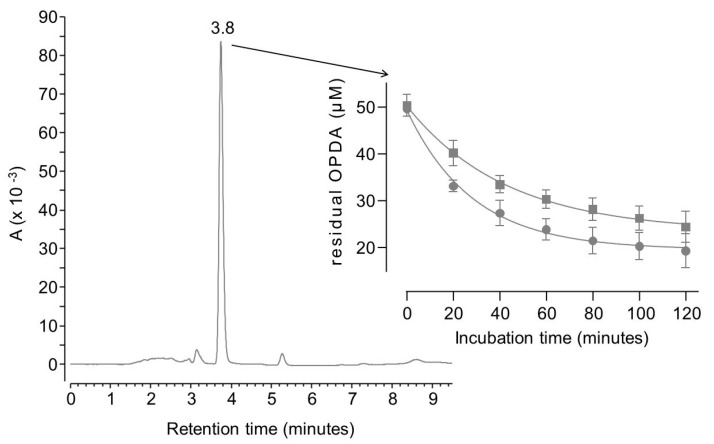
LC-UV chromatogram of 50 µM OPDA and plot of the residual concentration of OPDA (means ± standard deviation of triplicates) upon incubation with 30 µM MGO (circles) or BGO (squares).

As reported in Figure 1, when ortho-phenylenediamine (OPDA) is analyzed by the chromatographic method reported in Section 3.4.1, a single peak is detectable at 3.8 min. The residual concentration of OPDA decreases over time upon incubation with either MGO or BGO.

**Figure 2:**
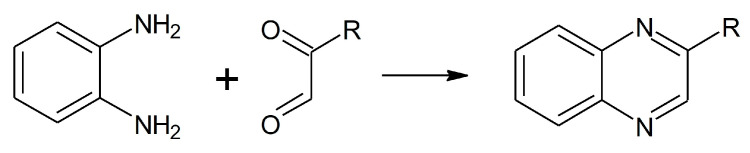
Reaction of OPDA with dicarbonyls (R = H glyoxal; R = CH_3_ methylglyoxal; R = CH_2_(C_6_H_5_) benzylglyoxal).

Both reactions were complete within two hours since residual OPDA reached a stable concentration of 20 µM. According to the reaction scheme reported in Figure 2, one mole of OPDA is expected to react with an equimolar amount of MGO or BGO to produce 2-methylquinoxaline or 2-benzylquinoxaline, respectively [25,27].

**Figure 3:**
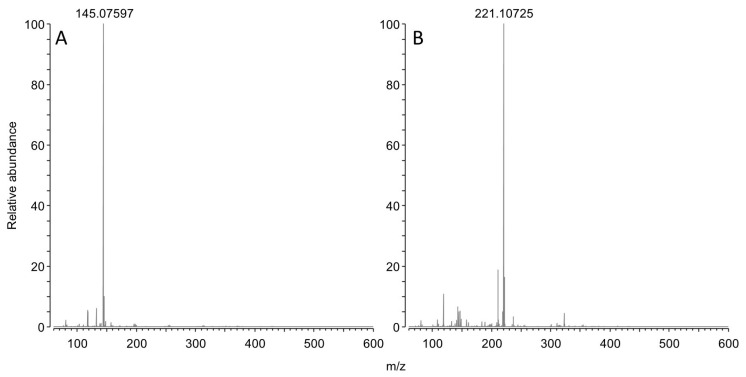
MS spectrum of OPDA upon 3 h incubation with an equimolar amount of MGO (**A**) or BGO (**B**).

This reaction scheme is consistent with the residual concentration of OPDA at the kinetics endpoints, since a molar excess of OPDA was used (i.e., 50 µM OPDA; 30 µM of either GO or MGO). The reaction scheme is also consistent with parallel ESI-MS experiments. Specifically, OPDA incubated with MGO produced a signal at 145.07597 *m*/*z* (Figure 3A), which is −0.4 ppm shy from the expected *m*/*z* value for monoprotonated 2-methylquinoxaline. Similarly, incubation with BGO produced a signal at 221.10725 *m*/*z* (Figure 3B), which is −0.3 ppm shy from the expected *m*/*z* value for monoprotonated 2 benzyl quinoxaline.

No trace of adducts with residual acetals of MGO or BGO, nor with any other impurities, were found during MS analyses. Therefore, BGO acetal, synthesized as reported in Section 3.2, was obtained as a pure compound and acetal hydrolysis, reported in Section 3.3, was completed for both MGO and BGO.

Kinetics data, shown in Figure 1, fit with a one-phase exponential decay, although extra sum-of-squares F test rejects the null hypothesis that the preferred model to fit all data is one curve. Separate fitting was then performed for data collected using BGO or MGO. The half-life of OPDA was found to be 20 min when it reacts with MGO, whereas a half-life of 30 min was found in parallel experiments with BGO. This confirms that MGO is about 1.5 as reactive as BGO towards OPDA.

**Figure 4:**
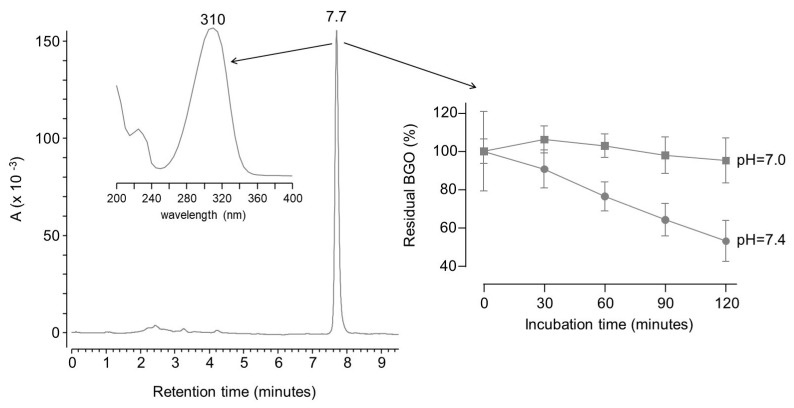
LC-UV chromatogram of 50 µM BGO and plot of the residual concentration of BGO (means ± standard deviation of triplicates) upon incubation in phosphate buffer at pH = 7 (squares) or pH = 7.4 (circles).

However, BGO gives a single peak chromatogram at around 7.7 min when analyzed by the LC method used to analyze OPDA. The peak was detectable since BGO has a characteristic UV maximum absorbance at 310 nm (see Figure 4).

On the contrary, no peak can be obtained in the same conditions for MGO or any other naturally occurring dicarbonyl. This is because such molecules have no strong chromophore, nor can they be easily separated by reversed phase chromatography owing to their high hydrophilicity. Since BGO compares with MGO in terms of reactivity towards OPDA, it can be used as surrogate of naturally occurring dicarbonyls (e.g., glyoxal, methylglyoxal) in order to set up an assay for the screening of dicarbonyl binders.

This assay resembles an LC-UV method that uses HNE as molecular probe to identify hit compounds able to react with α,β-unsaturated aldehydes. Such a method was successfully applied to study the properties of molecules such as carnosine, pyridoxamine, aminoguanidine, and hydralazine. Such molecules were also tested for their reactivity towards MGO, but the test required the setup of different assays using higher concentration of the reagents [23].

By using BGO as a molecular probe, it was possible to set up an LC-UV method for the screening of dicarbonyl binders with the same conditions reported in the literature for the screening of HNE binders. Unlike for the HNE assay, the reaction had to be performed at pH 7 instead of 7.4, since even slightly basic conditions induced a spontaneous degradation of BGO (see Figure 4). This was confirmed by two-way ANOVA test on the residual concentration of BGO. The test rejected the null hypothesis that the amount of BGO remains the same upon incubation in phosphate buffer at pH 7.4 for 60 min or more. On the contrary, the hypothesis was not rejected for experiments at pH 7 within 2 h. This is not surprising since dicarbonyls can undergo benzilic acid rearrangement at basic pH [23,29,30].

**Figure 5:**
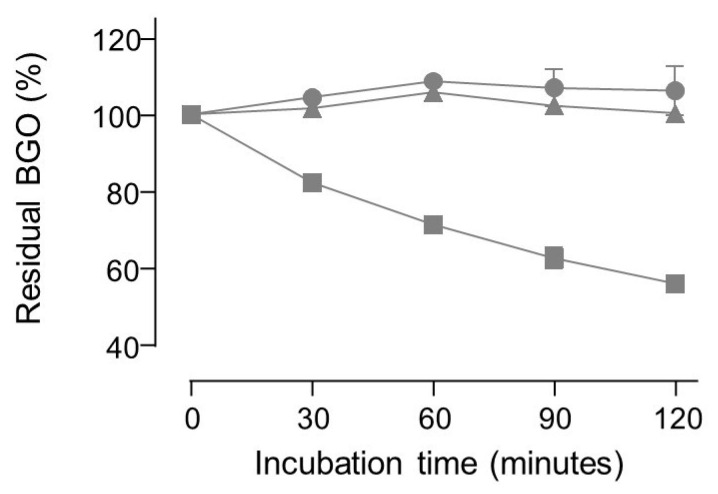
Plot of the residual concentration of 50 µM BGO (means ± standard deviation of triplicates) upon incubation in phosphate buffer at pH = 7 with 1 mM carnosine (circles) or 1 mM pyridoxamine (triangles) or 1 mM aminoguanidine (squares).

No apparent degradation of BGO was observed in neutral buffered solutions even when carnosine or pyridoxamine were added, whereas aminoguanidine induced a decrease in BGO concentration over time (see Figure 5).

Two-way ANOVA test on the residual concentration of BGO rejected the null hypothesis that the amount of BGO remains the same upon incubation with aminoguanidine for 30 min or more. On the contrary, the hypothesis was not rejected for experiments where aminoguanidine was replaced with either carnosine or pyridoxamine.

Incubation with hydralazine produced a very fast decrease in BGO peak area since less than 0.1% of the initial amount was detectable at 30 min. Immediately after spiking hydralazine into the BGO solution, the peak area was already below 20% of a reference sample (i.e., 50 µM BGO). ESI-MS experiments confirmed that no adducts between BGO and carnosine or pyridoxamine were detectable.

As for OPDA experiments, BGO produced adducts detectable by ESI-MS with both aminoguanidine and hydralazine. ESI-MS experiments also indicated that MGO and BGO reacts by the same mechanism already reported in the literature [23]. This feature is important since aryl derivatives such as phenylglyoxal (PGO) have been also considered as potential molecular probes during the design of the assay. However, BGO was synthesized to have the smallest compound containing a good chromophore for UV detection. On the contrary, PGO or other aryl derivatives were not considered since PGO was reported to have a different reactivity when compared to MGO and GO [31,32]. Moreover, aryl dicarbonyls are potentially more unstable due to a faster benzilic acid rearrangement [30].

The reactivity of the tested compounds towards BGO is partly consistent with the literature. Specifically, both aminoguanidine and hydralazine have been already reported as dicarbonyl binders [23,33]. However, literature data suggest similar reaction kinetics with MGO, whereas the experiments with BGO suggest that reaction with dicarbonyls is significantly faster for hydralazine when compared to aminoguanidine. In addition, in relation to carnosine, the data in the literature were not consistent with the data collected using the BGO assay. Specifically, a mild reactivity of MGO with carnosine has been reported [23], while no reactivity with BGO was found by the LC-UV assay, nor ESI-MS signals of carnosine adducts with BGO or MGO. Such discrepancies can be attributed to the experimental designs. In fact, literature data showing a reactivity of carnosine with MGO have been collected after 24 h incubation at pH 7.4 with reagents in the millimolar concentration range [23]. Such conditions can be conducive to benzilic acid rearrangement [29]. The impact of such a side reaction was not investigated for the assay used in the literature, since the method was not designed to detect MGO or its degradation product deriving from benzilic acid rearrangement. Therefore, the results obtained by the new LC-UV assay using BGO at pH 7 look more reliable since the assay was optimized to minimize side reactions.

## 3. Materials and Methods

### 3.1. Chemicals

HPLC grade water (18 MΩ × cm) was purified with a Milli-Q water system (Millipore; Milan; Italy). HPLC grade solvents and all other chemicals were purchased from Sigma Aldrich (Merck Life Science, Milan, Italy). Benzylglyoxal diethylacetal was synthesized as reported in Section 3.2.

### 3.2. Synthesis of Benzylglyoxal Diethylacetal

**Figure 6:**
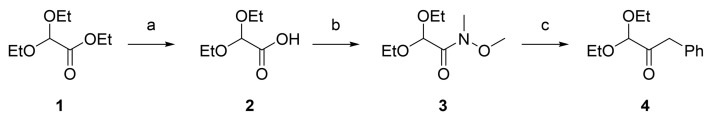
Synthesis of benzylglyoxal dimethylacetal: (a) 1N NaOH, EtOH, RT; (b) N-methylmorpholine, EDC·HCl, HOBT, N,O-dimethylhydroxylamine·HCl, DCM, RT; (c) BnMgBr, THF, 0 °C.

The general scheme of the procedures for the synthesis of benzylglyoxal diethylacetal is reported in Figure 6.

#### 3.2.1. Synthesis of 2,2-Diethoxyacetic Acid (Compound **2**, Figure 6)

Compound **2** was synthesized according to a modified reported procedure [34]. To begin, 1 N NaOH (2.8 mL) was added to a solution of ethyl 2,2-diethoxyacetate (compound **1**, Figure 1, 0.5 mL, 2.80 mmol) in ethanol (1.5 mL). The reaction mixture was stirred for 1 h at room temperature. The organic solvent was evaporated under a vacuum and the aqueous phase was extracted with diethyl ether (2 mL). The aqueous layer was then acidified and extracted with ethyl acetate (4 × 5 mL) and the combined organic layer was dried over anhydrous Na_2_SO_4_. The solvent was removed in vacuo to give compound **2** (403 mg, 2.72 mmol) as a clear oil. Yield: 97.3%. TLC (DCM/MeOH 7:3 + 1% formic acid) Rf: 0.63 ^1^H NMR (300 MHz, CDCl_3_) δ 4.96 (s, 1H), 3.78–3.63 (m, 4H), 1.27 (t, J = 7.1 Hz, 6H). NMR spectral data are consistent with those available in the literature [34].

#### 3.2.2. Synthesis of 2,2-Diethoxy-N-methoxy-N-methyl-acetamide (Compound **3**, Figure 6)

To a stirred solution of compound **2** (403 mg, 2.72 mmol) in DCM (10 mL) at 0 °C, N-methylmorpholine (0.45 mL, 4.08 mmol), EDC hydrochloride (548 mg, 2.86 mmol), and HOBT (438 mg, 2.86 mmol) were added. Then, N,O-dimethylhydroxylamine hydrochloride (292 mg, 2.99 mmol) was added to the solution. The reaction mixture was stirred at room temperature overnight. Afterward, the solvent was evaporated in vacuo, the crude was dissolved in diethyl ether (10 mL), then washed with a 10% aqueous solution of HCl (3 mL) and with a 10% aqueous solution of NaHCO_3_ (2 × 3 mL). The organic layer was dried over anhydrous Na_2_SO_4_, and the solvent was removed in vacuo to give compound **3** (300 mg, 1.57 mmol) as a clear oil. Yield: 57.7%. TLC (cyclohexane: ethyl acetate 8:2) Rf: 0.27 ^1^H NMR (300 MHz, CDCl_3_) δ 5.33 (bs, 1H), 3.80–3.58 (m, 5H), 3.47 (q, J = 7.0 Hz, 2H), 3.21 (bs, 3H), 1.25 (t, J = 7.1 Hz, 3H), 1.22–1.17 (m, 3H).

#### 3.2.3. Synthesis of Benzylglyoxal Diethylacetal (Compound **4**, Figure 6)

Benzylmagnesiumbromide was freshly prepared from benzylbromide (0.56 mL, 4.71 mmol) and magnesium (1.72 gr, 70.65 mmol) in tetrahydrofuran (7.5 mL) Then, it was added under vigorous stirring to a solution of compound **3** (300 mg, 1.57 mmol) in tetrahydrofuran (5 mL). The reaction was stirred for 2 h at 0 °C. The reaction was quenched with 20 mL of a 10% aqueous solution of HCl, and the aqueous layer was extracted with ethyl acetate (2 × 20 mL). Afterward, the combined organic phases were sequentially washed with brine, dried over anhydrous Na_2_SO_4_, filtered, and concentrated under reduced pressure, affording a crude that was purified by flash chromatography on silica gel (cyclohexane/dichloromethane 6:4). The pure product compound **4** (187 mg, 0.84 mmol) was isolated as a colorless oil. Yield: 53.6%. TLC (cyclohexane: ethyl acetate 8:2) Rf: 0.81 ^1^H NMR (300 MHz, CDCl_3_) δ 7.39–7.17 (m, 5H), 4.64 (s, 1H), 3.90 (s, 2H), 3.81–3.64 (m, 2H), 3.63–3.45 (m, 2H), 1.25 (t, J = 7.0 Hz, 6H). 13C NMR (75 MHz, CDCl_3_) δ 203.13, 133.76, 129.74, 128.44, 126.81, 102.34, 63.38, 43.65, 26.91, 15.14. NMR spectral data are consistent with those available in the literature [35].

### 3.3. Stock Solutions for Chromatographic and Mass Spectrometric Analyses

Titrated stock solutions of methylglyoxal (MGO) and benzylglyoxal (BGO) were obtained by acidic hydrolysis of the corresponding acetals. To provide the best hydrolysis yield the reaction was performed at 60 °C for 5 h in 1 M hydrochloric acid containing 50% of acetonitrile to ensure solubilization of BGO. The hydrolysis yield and stock solution concentration were determined indirectly by using a chromatographic assay to determine the residual amount of OPDA (see Section 3.4.1). Briefly, an aliquot was picked up from the reaction batch and diluted a hundred-fold in 100 mM phosphate buffer pH 7 containing 50% acetonitrile and a molar excess of OPDA. The reaction with OPDA provides derivatization of dicarbonyls to produce 2-substituted quinoxaline derivatives, as seen in the reaction scheme reported in Figure 2 [25,27]. The moles of MGO or BGO can be then calculated as the difference between the moles of OPDA added and the residual moles at the end of the incubation.

### 3.4. Methods

#### 3.4.1. OPDA Binding Kinetics

The analyses were provided by a Surveyor HPLC system equipped with a Gemini C18 column (250 × 2.1 mm, 5 μM particle size, 110 Å pore size, Phenomenex, Milan, Italy). An Optisolv mini filter 0.5 mm (Sigma) was installed before the column to protect it from particulates. A solution of OPDA (50 µM in 100 mM phosphate buffer pH 7 containing 50% acetonitrile) was spiked with MGO or BGO down to 30 µM final concentration and incubated into the autosampler at 37 °C. Experiments were conducted in triplicates and aliquots of 3 µL of all samples were picked up at different times and injected into the column. The elution of the analytes was performed at 30 °C by a 200 µL/min flowrate with the mobile phase reported in Table 1.

UV detection within a wavelength range from 200 to 400 nm was provided by a photodiode array detector. The residual concentration of OPDA was measured by using a calibration curve, and was prepared from the analysis of OPDA standards. Calibration curve standards were prepared in triplicates by dilution of the OPDA stock solution down to concentrations ranging from 1 to 60 µM. Peak areas of chromatograms were extracted from single wavelength profiles at 290 nm. Instrument control and data analysis were provided by the Xcalibur 2.07 software (Thermo Fisher Scientific, Rodano, MI, Italy).

#### 3.4.2. Direct Determination of Dicarbonyl Binding Activity

Dicarbonyl binding activity was determined by measuring the residual amount of BGO over time by means of LC-UV. The residual concentration of BGO was measured by using a calibration curve prepared from the analysis of BGO standards. Calibration curve standards were prepared in triplicates by dilution of the BGO stock solution down to concentrations ranging from 1 to 60 µM. Peak areas of chromatograms were extracted from single wavelength profiles at 310 nm. For each tested compound a stock solution was prepared in water or the most appropriate solvent to ensure solubilization.

The compounds were then separately spiked into 100 mM phosphate buffer containing 10% acetonitrile and 50 µM BGO down to 1 mM final concentration and incubated into the autosampler at 37 °C. The experiments were conducted in triplicate and the analyses were performed by using the same procedure reported in Section 3.4.1.

#### 3.4.3. Mass Spectrometry

The samples were diluted tenfold in 30% aqueous acetonitrile containing 0.1% formic acid and infused at 5 µL/min flowrate directly into a Finnigan Ion Max 2 electrospray (ESI) by using a glass microliter syringe. Sample nebulization and ionization were provided by using a metal capillary 140 mm long and with a 160 µm inner diameter, and applying a spray voltage of 5 kV, 20 units of sheath gas (nitrogen), and a capillary temperature of 275 °C. MS spectra were collected by an LTQ-Orbitrap™ XL-ETD mass spectrometer within a scan range from 60 to 600 *m*/*z*. The analyzer was working on 5 × 10^5^ ion per scan, with a resolution of 100,000 (full width at half-maximum at 400 *m*/*z*). Lock mass option was enabled to provide real time internal mass calibration. Reference masses were 20 ions identified as background ESI trace contaminants and already listed as common mass spectrometry contaminants [36]. Instrument control was provided by the software LTQ Tune plus 2.5.5 and data extraction and analysis by Xcalibur 2.1 (Thermo Fisher Scientific, Rodano, MI, Italy).

#### 3.4.4. NMR Spectroscopy

^1^H-NMR spectra were recorded using an FT-spectrometer operating at 300 MHz while ^13^C-NMR were recorded at 75.43 MHz. Chemical shifts are reported in ppm relative to the residual solvent (CHCl_3_) as an internal standard. Signal multiplicity is designed according to the following abbreviations: s = singlet, d = doublet, dd = doublet of doublets, t = triplet, m = multiplet, and bs = broad singlet. Purifications were performed by flash chromatography using silica gel (particle size 40–63 μm, Merck) on Isolera^TM^ (Biotage, Uppsala, Sweden) apparatus.

#### 3.4.5. Data Analysis and Statistics

Fitting of calibration curves for BGO and OPDA quantification were performed by using the simplest model describing the relationship between peak area and amount injected. A straight line with least squares fit applying no weighting was found to be the simplest model for all calibration curves and was applied to interpolate the concentrations of samples. A one phase exponential decay with least squares fit applying no weighting was found to be the simplest model for describing the time-dependent decrease of OPDA concentration when the compound was incubated with either BO or MGO. Extra sum-of-squares F test was used to compare the different models and to assess whether a single curve fits all dataset. Two-way ANOVA test on the residual concentration of BGO was used to compare the residual concentration across the experiments conducted in different conditions (e.g., pH of the solutions, compounds incubated with BGO). Calibration curves fittings, one phase exponential decay fitting, and two-way ANOVA were performed by Prism (version 9, GraphPad software LLC).

## 4. Conclusions

BGO can be considered as a convenient surrogate of naturally occurring dicarbonyls, as it retains the same reaction mechanism and compares with them in terms of kinetics. In addition, it can be directly analyzed by reversed phase chromatography with an UV detector without derivatization. Such features make it a good molecular probe for the set up of a simple and cost-effective chromatographic assay for the identification of hit compounds able to bind dicarbonyls. The use of BGO as a probe allows one to quantify the extent of the reaction by monitoring the decrease of BGO, instead of by monitoring the increase of the product of the reaction. This has multiple advantages. First, the method can be used even if the tested compound or its reaction products are not responsive to specific detectors. Second, the same method can be used for quantification of different substrates, which is not the case if the reaction product is monitored instead, because separate calibration curves are required for each compound. Such a method parallels other known LC-UV assays that were built for the identification of hit compounds able to bind other classes of naturally occurring carbonyl compounds (i.e., α,β-unsaturated aldehydes). The assay described herein can make a significant impact in pharmaceutical research, especially for the characterization of the selectivity profile of candidate drugs and preservatives aimed at detoxifying specific aldehyde classes that are involved in cell damage and human disease.

## Figures and Tables

**Table 1:** LC-UV gradient program.

Time (Minutes)	10 mM NH_4_Cl	CH_3_CN
0.00	70%	30%
1.00	70%	30%
6.00	40%	60%
6.00	70%	30%
9.50	70%	30%
22.00	95%	5%

## Data Availability

The data presented in this study are available on request from the corresponding author.

## Sample Availability

Samples of the compounds are not available from the authors.

## References

[1] Arribas-Lorenzo G., Morales F.J. (2010). Analysis, distribution, and dietary exposure of glyoxal and methylglyoxal in cookies and their relationship with other heat-induced contaminants. J. Agric. Food Chem..

[2] Kwak S., Choi Y.S., Na H.G., Bae C.H., Song S.Y., Kim Y.D. (2021). Glyoxal and Methylglyoxal as E-cigarette Vapor Ingredients-Induced Pro-Inflammatory Cytokine and Mucins Expression in Human Nasal Epithelial Cells. Am. J. Rhinol. Allergy.

[3] Wang Y., Ho C.T. (2012). Flavour chemistry of methylglyoxal and glyoxal. Chem. Soc. Rev..

[4] Yamaguchi M., Ishida J., Xuan Z.X., Nakamura A., Yoshitake T. (1994). Determination of Glyoxal, Methylglyoxal, Diacethyl, and 2, 3-Pentanedione in Fermented Foods by High-Performance Liquid Chromatography with Fluorescence Detection. J. Liq. Chromatogr..

[5] Lange J.N., Wood K.D., Knight J., Assimos D.G., Holmes R.P. (2012). Glyoxal formation and its role in endogenous oxalate synthesis. Adv. Urol..

[6] Thornalley P.J., Langborg A., Minhas H.S. (1999). Formation of glyoxal, methylglyoxal and 3-deoxyglucosone in the glycation of proteins by glucose. Biochem. J..

[7] Chakraborty S., Karmakar K., Chakravortty D. (2014). Cells producing their own nemesis: Understanding methylglyoxal metabolism. Iubmb Life.

[8] Phillips S.A., Thornalley P.J. (1993). The formation of methylglyoxal from triose phosphates. Investigation using a specific assay for methylglyoxal. Eur. J. Biochem..

[9] Rabbani N., Xue M., Thornalley P.J. (2016). Dicarbonyls and glyoxalase in disease mechanisms and clinical therapeutics. Glycoconj J..

[10] Izaguirre G., Kikonyogo A., Pietruszko R. (1998). Methylglyoxal as substrate and inhibitor of human aldehyde dehydrogenase: Comparison of kinetic properties among the three isozymes. Comp. Biochem. Physiol. B Biochem. Mol. Biol..

[11] Vander Jagt D.L., Hunsaker L.A. (2003). Methylglyoxal metabolism and diabetic complications: Roles of aldose reductase, glyoxalase-I, betaine aldehyde dehydrogenase and 2-oxoaldehyde dehydrogenase. Chem. Biol. Interact..

[12] Vander Jagt D.L., Robinson B., Taylor K.K., Hunsaker L.A. (1992). Reduction of trioses by NADPH-dependent aldo-keto reductases. Aldose reductase, methylglyoxal, and diabetic complications. J. Biol. Chem..

[13] Reichard G.A., Skutches C.L., Hoeldtke R.D., Owen O.E. (1986). Acetone metabolism in humans during diabetic ketoacidosis. Diabetes.

[14] Abordo E.A., Minhas H.S., Thornalley P.J. (1999). Accumulation of alpha-oxoaldehydes during oxidative stress: A role in cytotoxicity. Biochem. Pharmacol..

[15] Lo T.W., Westwood M.E., McLellan A.C., Selwood T., Thornalley P.J. (1994). Binding and modification of proteins by methylglyoxal under physiological conditions. A kinetic and mechanistic study with N alpha-acetylarginine, N alpha-acetylcysteine, and N alpha-acetyllysine, and bovine serum albumin. J. Biol. Chem..

[16] Ahmed N., Thornalley P.J., Dawczynski J., Franke S., Strobel J., Stein G., Haik G.M. (2003). Methylglyoxal-derived hydroimidazolone advanced glycation end-products of human lens proteins. Investig. Ophthalmol. Vis. Sci..

[17] Li Y., Cohenford M.A., Dutta U., Dain J.A. (2008). The structural modification of DNA nucleosides by nonenzymatic glycation: An in vitro study based on the reactions of glyoxal and methylglyoxal with 2′-deoxyguanosine. Anal. Bioanal. Chem..

[18] Lai S.W.T., Lopez Gonzalez E.J., Zoukari T., Ki P., Shuck S.C. (2022). Methylglyoxal and Its Adducts: Induction, Repair, and Association with Disease. Chem. Res. Toxicol..

[19] Schalkwijk C.G., Stehouwer C.D.A. (2020). Methylglyoxal, a Highly Reactive Dicarbonyl Compound, in Diabetes, Its Vascular Complications, and Other Age-Related Diseases. Physiol. Rev..

[20] Wondrak G.T., Cervantes-Laurean D., Roberts M.J., Qasem J.G., Kim M., Jacobson E.L., Jacobson M.K. (2002). Identification of alpha-dicarbonyl scavengers for cellular protection against carbonyl stress. Biochem. Pharmacol..

[21] Tan D., Wang Y., Lo C.Y., Ho C.T. (2008). Methylglyoxal: Its presence and potential scavengers. Asia Pac. J. Clin. Nutr..

[22] Lobner J., Degen J., Henle T. (2015). Creatine is a scavenger for methylglyoxal under physiological conditions via formation of N-(4-methyl-5-oxo-1-imidazolin-2-yl)sarcosine (MG-HCr). J. Agric. Food Chem..

[23] Colzani M., De Maddis D., Casali G., Carini M., Vistoli G., Aldini G. (2016). Reactivity, Selectivity, and Reaction Mechanisms of Aminoguanidine, Hydralazine, Pyridoxamine, and Carnosine as Sequestering Agents of Reactive Carbonyl Species: A Comparative Study. ChemMedChem.

[24] Vistoli G., Orioli M., Pedretti A., Regazzoni L., Canevotti R., Negrisoli G., Carini M., Aldini G. (2009). Design, synthesis, and evaluation of carnosine derivatives as selective and efficient sequestering agents of cytotoxic reactive carbonyl species. ChemMedChem.

[25] Fritzsche S., Billig S., Rynek R., Abburi R., Tarakhovskaya E., Leuner O., Frolov A., Birkemeyer C. (2018). Derivatization of Methylglyoxal for LC-ESI-MS Analysis-Stability and Relative Sensitivity of Different Derivatives. Molecules.

[26] Li P., Zhu Y., He S., Fan J., Hu Q., Cao Y. (2012). Development and validation of a high-performance liquid chromatography method for the determination of diacetyl in beer using 4-nitro-o-phenylenediamine as the derivatization reagent. J. Agric. Food Chem..

[27] Barros A., Rodrigues J.A., Almeida P.J., Oliva-Teles M.T. (1999). Determination of Glyoxal, Methylglyoxal, and Diacetyl in Selected Beer and Wine, by Hplc with UV Spectrophotometric Detection, after Derivatization with o-Phenylenediamine. J. Liq. Chromatogr. Relat. Technol..

[28] Brun N., Gonzalez-Sanchez J.M., Demelas C., Clement J.L., Monod A. (2023). A fast and efficient method for the analysis of alpha-dicarbonyl compounds in aqueous solutions: Development and application. Chemosphere.

[29] Evans T.W., Dehn W.M. (1930). The Benzilic Acid Rearrangement. J. Am. Chem. Soc..

[30] Burke A.J., Marques C.S. (2007). Mechanistic and synthetic aspects rearrangements of the benzilic acid and ester. Mini-Rev. Org. Chem..

[31] Takahashi K. (1977). The reactions of phenylglyoxal and related reagents with amino acids. J. Biochem..

[32] Takahashi K. (1977). Further studies on the reactions of phenylglyoxal and related reagents with proteins. J. Biochem..

[33] Thornalley P.J. (1998). Glutathione-dependent detoxification of alpha-oxoaldehydes by the glyoxalase system: Involvement in disease mechanisms and antiproliferative activity of glyoxalase I inhibitors. Chem. Biol. Interact..

[34] Pinto A., Conti P., Grazioso G., Tamborini L., Madsen U., Nielsen B., De Micheli C. (2011). Synthesis of new isoxazoline-based acidic amino acids and investigation of their affinity and selectivity profile at ionotropic glutamate receptors. Eur. J. Med. Chem..

[35] Shi Y., Wang J., Yang F., Wang C., Zhang X., Chiu P., Yin Q. (2022). Direct asymmetric reductive amination of alpha-keto acetals: A platform for synthesizing diverse alpha-functionalized amines. Chem. Commun..

[36] Keller B.O., Sui J., Young A.B., Whittal R.M. (2008). Interferences and contaminants encountered in modern mass spectrometry. Anal. Chim. Acta.

